# C/EBPα Short-Activating RNA Suppresses Metastasis of Hepatocellular Carcinoma through Inhibiting EGFR/β-Catenin Signaling Mediated EMT

**DOI:** 10.1371/journal.pone.0153117

**Published:** 2016-04-06

**Authors:** Hongbo Huan, Xudong Wen, Xuejiao Chen, Lili Wu, Weihui Liu, Nagy A. Habib, Ping Bie, Feng Xia

**Affiliations:** 1 Institute of Hepatobiliary Surgery, Southwest Hospital, Third Military Medical University, Chongqing, China; 2 Laboratory of Biotherapy of Cancer, Institute of Pathology and Southwest Cancer Center, Southwest Hospital, Third Military Medical University, Chongqing, China; 3 General Surgery Center, Chengdu Military General Hospital, Chengdu, Sichuan Province, China; 4 Department of Surgery and Cancer, Faculty of Medicine, Imperial College London, London, United Kingdom; University of Navarra School of Medicine and Center for Applied Medical Research (CIMA), SPAIN

## Abstract

Hepatocellular carcinoma is associated with high mortality, and tumor metastasis is an important reason for poor prognosis. However, metastasis has not been effectively prevented in clinical therapy and the mechanisms underlying metastasis have not been fully characterized. CCAAT/enhancer-binding protein-α (C/EBPα) is a transcriptional regulator with an essential role in tumor metastasis. We used short-activating RNAs (saRNA) to enhance expression of C/EBPα. Intravenous injection of C/EBPα-saRNA in a nude mouse liver orthotopic xenograft tumor model inhibited intrahepatic and distant metastasis. C/EBPα-saRNA-treated mice showed increased serum levels of albumin and decreased alanine aminotransferase (ALT), glutamic-oxalacetic transaminase (AST), indicating a role of C/EBPα in improving liver function. Migration and invasion were inhibited in hepatoma cell lines transfected with C/EBPα-saRNA. We also observed an inhibition of epithelial-mesenchymal transition (EMT) and suppression of epidermal growth factor receptor (EGFR), EGFR phosphorylation, and β-catenin in C/EBPa-saRNA-transfected cells. Our results suggested that C/EBPα-saRNA successfully inhibited HCC metastasis by inhibiting EGFR/β-catenin signaling pathway mediated EMT *in vitro* and *in vivo*.

## Introduction

Liver cancer is the fifth most frequent cancer and the second leading cause of cancer death worldwide in men [1]. An estimated 422100 people die from liver cancer and more than 466100 new cases are diagnosed each year in China [2]. Hepatocellular carcinoma (HCC) is the most common primary liver cancer, and high frequencies of postoperative recurrence and metastasis mainly result in the poor prognosis [3]. Thus, better understanding of the molecular mechanisms of tumor initiation, progression, and metastasis is required for the identification of new therapeutic targets and development of effective treatment strategies [4].

Several studies have demonstrated an involvement of epithelial-mesenchymal transition (EMT) [5], liver cancer stem cells [6], and non-coding RNA [7] in HCC metastasis. However, the exact etiology of hepatoma cell migration and invasion has not yet been fully uncovered. CCAAT/enhancer-binding protein-α (C/EBPα) is a transcriptional regulator that plays both positive and negative transcriptional regulatory roles in normal physiological processes and disease occurrence, which is highly expressed in liver, lung, peripheral blood mononuclear cells and placenta [8]. C/EBPα generally acts as a tumor suppressor and is involved in regulating cancer cell differentiation, proliferation, apoptosis and metabolism. Low expression of C/EBPα has been identified in several types of cancer cells [9].

Recent reports have shown that C/EBPα is widely involved in invasion and metastasis in multiple tumor types, including gastric cancer [10], breast cancer [11], liver [12] and bladder cancer [13]. Reebye et al. [14] generated short-activating RNAs (saRNA) molecules to upregulate transcript levels of C/EBPα and reported reduced tumor burden and improved liver function in a liver cirrhosis/HCC model. Though C/EBPα-saRNA could inhibit tumor growth, whether it contributes to metastasis is uncertain. Therefore, in this study we examined whether C/EBPα-saRNA could regulate tumor metastasis in a nude mouse liver orthotopic xenograft tumor model.

## Materials and Methods

### Cell culture and chemicals

HepG2 and SMMC-7721 HCC cells were purchased from ATCC (Rockefeller, MD, USA) and maintained in complete dulbecco’s modified eagle’s medium (DMEM) (Gibco, Grand Island, NY, USA) supplemented with 10% fetal bovine serum (Gibco), 100 U/mL of penicillin and 100 μg/mL of streptomycin at 37°C in 5% CO_2_. HepG2 cells were infected with Lv-PGK3-RFP lentivirus to generate HepG2-RFP cells for *in vivo* imaging. Trypsin-EDTA (0.05%) (Life Technologies, Carlsbad, CA, USA) was used for cell passages. Short-activating RNAs was kindly provided by Professor Habib, Department of Surgery and Cancer, Faculty of Medicine, Imperial College London.

### Animals

Twenty-five male Balb/c nude mouse, aged 7 weeks old and weighing 18–22 g were purchased from the Institute of Experimental Animals in the Third Military Medical University (China; rodent license no. SYXK (yu) 2012–0002). All mice were maintained on a 12-h/12-h light/dark cycle with free access to standard laboratory feed and water. In order to reduce the suffering in mice, all surgery and *in vivo* imaging were performed under sodium pentobarbital anesthesia. All procedures performed in studies involving animals were in accordance with the Institutional Animal Care and Use Committee of the Third Military University (China), and were approved by the Animal Research Ethics Committee of the Third Military University.

### Transfection of saRNA

Transfection of scramble-saRNA and C/EBPα-saRNA into hepatoma cells was performed using TransMessenger™ Transfection Reagent (Qiagen, Germany). Briefly, dilute 4 μl Enhancer R in Buffer EC, and add 2 μg saRNA. The final volume should be 100 μl. Incubate at room temperature (15–25°C) for 5 min. Then add 8 μl TransMessenger Transfection Reagent to the mixture, and incubate the samples for 10 min at room temperature to allow transfection-complex formation. While complex formation takes place, aspirate the growth medium from the plate, and carefully wash cells once with 3 ml PBS. We then added 900 μl growth medium without serum or antibiotics to the tube containing the transfection complexes and added the entire mixture to cells and incubated cells with the transfection complexes for 3 h under their normal growth conditions. Remove the complexes from the cells and wash cells once with PBS, and then add 2 ml fresh medium containing serum and antibiotics to the cells, and incubate cells under their normal growth conditions to allow protein expression.

### Liver orthotopic xenograft tumor model and *in vivo* imaging

HepG2-RFP cells were harvested by trypsinization, washed three times with cold serum-free medium and resuspended with serum-free DMEM medium. Nude mice were anesthetized with intraperitoneal injection of 1% pentobarbital sodium (Sigma-Aldrich; 100 mL/kg body weight) and a longitudinal abdominal incision was made to visualize the liver. HepG2-RFP cancer cells (1.0×10^5^) were injected below the liver capsule of nude mice. Scramble or C/EBPα-saRNA-PAMAM was intravenously injected respectively per 24 h. For *in vivo* imaging based on fluorescent proteins, the IVIS Spectrum Pre-clinical *In Vivo* Imaging System (PerkinElmer, Waltham, MA, USA) was used. Primary tumors and metastases were collected for subsequent analyses.

### Histological assessment

Liver and lung tissues were directly fixed in OCT Compound, frozen at −80°C, and sectioned at 5 μm thickness using a freezing microtome (Leica, Barnack, Germany). The sections were stained with a Hematoxylin & Eosin Staining Kit (Beyotime, Shanghai, China). To detect the expression of epidermal growth factor receptor (EGFR), and β-catenin in liver tissue, staining was respectively performed using antibody incubated at 4°C overnight. Slides were rinsed in PBS and the immunoreactive were visualized by a DAKO EnVision Detection System and counterstained with hematoxylin.

### Liver function analysis

Mouse serum samples collected immediately after euthanization were measured for albumin, alanine aminotransferase (ALT) and glutamic-oxalacetic transaminase (AST) levels by Automatic Biochemical Analyzer (AU5800, Beckman Coulter Inc., CA, USA).

### Real-time quantitative reverse transcription-PCR

Reverse transcription-PCR was performed to determine the expression of albumin, C/EBPα, c-Myc, Axis inhibition protein 2 (Axin2), Cyclin D1 (Ccnd 1), and leucine-rich repeat-containing G-protein coupled receptor 5 (Lgr5) genes. Reverse transcription was performed using an RT-core kit (Takara) and quantitative PCR (qPCR) was performed using the qPCR Mastermix for SYBRGreen I (Takara) according to the manufacturer’s instructions. Primers are shown in Table 1. Relative quantification was performed using the standard curve method with a three-step dilution of pooled sample cDNA standards as a reference. The percentage change in expression for each gene between groups was determined by calculating the ratio to the internal reference gene.

**Table 1:** Primers and product size.

Gene	Primers(5’-3’)	Product Size(bp)
h-C/EBPα-F*	AACACGAAGCACGATCAGTCC	211
h-C/EBPα-R*	CTCATTTTGGCAAGTATCCGA	
h-Albumin-F	TCAGCCTTGCAGCACTTCTCTACA	254
h-Albumin-R	ACAGAATCCTTGGTGAACAGGCGA	
h-c-Myc-F	CCTCCACTCGGAAGGACTATC	137
h-c-Myc-R	TGTTCGCCTCTTGACATTCTC	
h-Axin2-F	CTCCTTATCGTGTGGGCAGT	240
h-Axin2-R	CTTCATCCTCTCGGATCTGC	
h-Ccnd1-F	CCTGTCCTACTACCGCCTCA	175
h-Ccnd1-R	CACCTCCTCCTCCTCCTCTT	
h-Lgr5-F	CTCCCAGGTCTGGTGTGTTG	149
h-Lgr5-R	GAGGTCTAGGTAGGAGGTGAAG	
h-GAPDH-F	AGGGGCCATCCACAGTCTTC	258
h-GAPDH-R	AGAAGGCTGGGGCTCATTTG	
m-CEBPA-F	GAGGGACTGGAGTTATGACAAG	111
m-CEBP-R	TGCACACTGCCATTGCACAA	
m-GAPDH-F	AGAAGGCCGGGGCCCACTTG	258
m-GAPDH-R	AGGGGCCATCCACAGTCTTC	

*F: forward, R: reverse. h: Human; m: Mouse.

### Cell migration and invasion assays

Matrigel (2 μg/well; BD Biosciences, San Jose, CA, USA) was used to pre-coat 24 well plates with 8-μm pore size polycarbonate membrane (Millipore, MA, USA) for invasion assays. Cells transfected with saRNA (8.0×10^4^) were seeded on the upper chamber with serum-free DMEM (200μl). DMEM (1 mL) with 30% serum was added to the lower chamber. After 18 h, cell migration or invasion (plates coated with Matrigel) was terminated and the membranes were fixed with 4% paraformaldehyde and stained with Crystal Violet. Five visual fields were randomly selected from each membrane, and the cell numbers were counted via a light microscope. All experiments were performed in triplicate.

### Wound healing assay

HepG2 and SMMC-7721 cells transfected with scramble or C/EBPα-saRNA were plated in 6 well plates in duplicate at ~80% confluency (approximately 48 h). A linear wound was created by dragging a 10 μl pipette tip through the cell monolayer. The cells were washed with PBS and then complete media was added. At 0, 12, 24 h post-wounding, cells were examined under an Olympus inverted microscope.

### Western blot

Protein samples from cell lines or liver tissues were obtained using Protein Extraction Reagent (Thermo Fisher Scientific). The protein samples were separated by SDS-PAGE and transferred to nitrocellulose membranes. Membranes were blocked for 2 h at room temperature and incubated overnight at 4°C with primary antibodies (1:1000). Antibodies against E-cadherin, ADAM metallopeptidase domain 17 (ADAM17) and C/EBPα were purchased from Abcam; N-Cadherin, Vimentin, and Slug antibodies were purchased from Epitomics; albumin and β-catenin antibodies were from Santa Cruz Biotechnology; EGFR and phospho-EGF receptor antibodies were from Cell Signaling Technology. Membranes were washed and incubated for 2 h with secondary antibodies (Sigma-Aldrich), followed by processing with Clarity Western ECL Substrate (Bio-Rad Laboratories Co. Ltd.). Band intensities were measured by Image Lab software (5.2.1 Version, Bio-Rad Laboratories Co. Ltd.).

### Statistical analysis

Statistical analysis was performed using IBM SPSS Statistics 22 software (IBM, USA). Analysis of variance followed by a Student’s t-test was used to compare the control and test groups. The qRT-PCR data were analyzed using parametric frequentist statistics. Survival analysis was modeled using Kaplan–Meier plots. All data are expressed as means ± SD. All tests were two-sided. Values of P < 0.05 were considered to indicate statistical significance.

## Results

### Intravenous injection of C/EBPα-saRNA-dendrimer in nude mouse with transplanted tumor results in inhibited tumor metastasis and improved liver function

**Fig 1:**
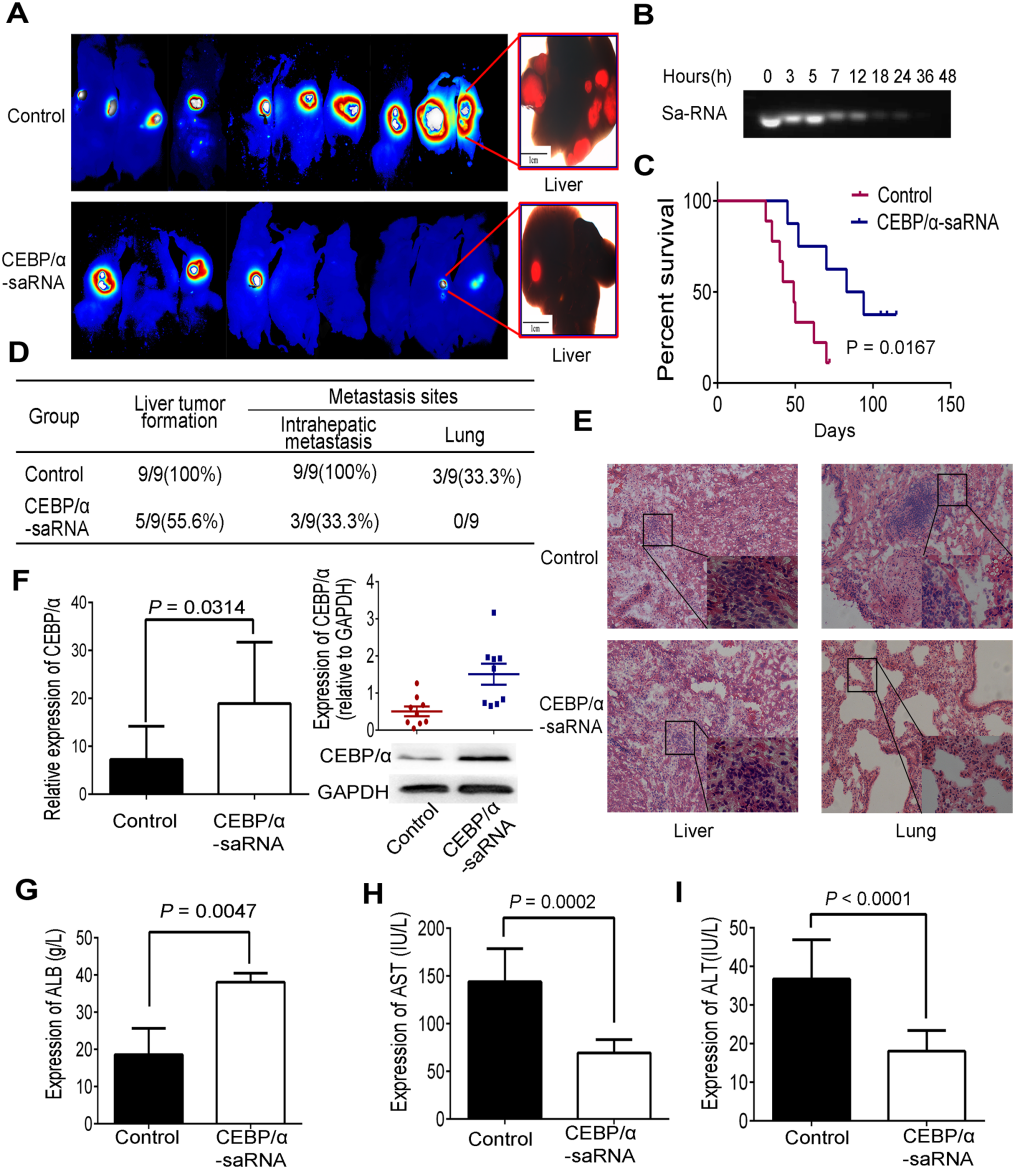
Intravenous injection of C/EBPα-saRNA-dendrimer inhibited tumor metastasis and improved liver function in nude mice with tumors from HepG2-RFP cells. (A) Imaging of liver orthotopic xenograft tumors after injection of C/EBPα-saRNA on day 40 post-injection of HepG2-RFP cells and scramble-saRNA as the control. Higher fluorescence intensity and intrahepatic metastasis was observed in the control group. (B) C/EBPα-saRNA-dendrimer was tested for nuclease sensitivity in nude mice for indicated times. (C) Survival analysis of C/EBPα-saRNA-injected mouse groups and the control (n = 9) (log rate: 0.0167). (D) Statistical table of tumor formation, intrahepatic and distant metastasis. (E) Hematoxylin and eosin staining of liver and lung from C/EBPα-saRNA injected mice and the control. (F) Expression of C/EBPα was determined by RT-PCR and western blotting in liver tissue of mice injected with C/EBPα-saRNA-dendrimer or the control. (G-I) C/EBPα-saRNA-dendrimer injected nude mice showed significant changes in circulating levels of serum albumin (G), AST (H) and ALT (I). Data represent mean±SD.

C/EBPα, as a transcription factor, exhibits essential role in the progress of tumor. To understand the role of C/EBPα-saRNA in liver tumor metastasis, we established a liver orthotopic xenograft tumor model in nude mice. *In vivo* imaging of the mice over 40 days revealed that the size of the tumor (fluorescence intensity) and intrahepatic metastasis in C/EBPα-saRNA-dendrimer-treated mice were significantly lower than that of scramble-saRNA-dendrimer group (Fig 1A). The stability of C/EBPα-saRNA was then tested by performing nuclease activity assays using blood samples from C/EBPα-saRNA-dendrimer-treated nude mice, and found almost complete degradation of C/EBPα-saRNA by 24 h (Fig 1B). Thus, we injected nude mice once a day. Survival analysis revealed a longer survival time of the C/EBPα-saRNA-dendrimer-treated mouse and log rate was 0.0167 (Fig 1C). To verify intrahepatic and distant metastasis, liver and lung organs were examined. The liver tumor formation rate (55.6%), intrahepatic metastasis (33.3%) and lung tumor formation rate (none) were lower in the C/EBPα-saRNA compared with the scramble-saRNA treatment group (100%, 100%, and 33.3%, respectively) (Fig 1D). Hematoxylin and eosin staining showed that cellular atypia and hyperchromasia in hepatic nodules were found in both groups, but metastasis lesions were only found in the lung in the scramble-saRNA-dendrimer group (Fig 1E). Upon trasnfection with C/EBPα-saRNA, the expression of C/EBPα in transcript (p = 0.0314) and protein levels (p = 0.004) were significantly high in C/EBPα-saRNA-dendrimer compared with the scramble-saRNA-dendrimer group (Fig 1F). Intravenous injection of C/EBPα-saRNA-dendrimer in mouse improved their liver function, with a corresponding improvement of albumin and decline of AST and ALT (Fig 1G–1I). It was also observed in male rats with liver cirrhosis and HCC [14].

### Transfection of C/EBPα-saRNA in hepatoma cell lines inhibits cell migration and invasion by suppression of EMT

**Fig 2:**
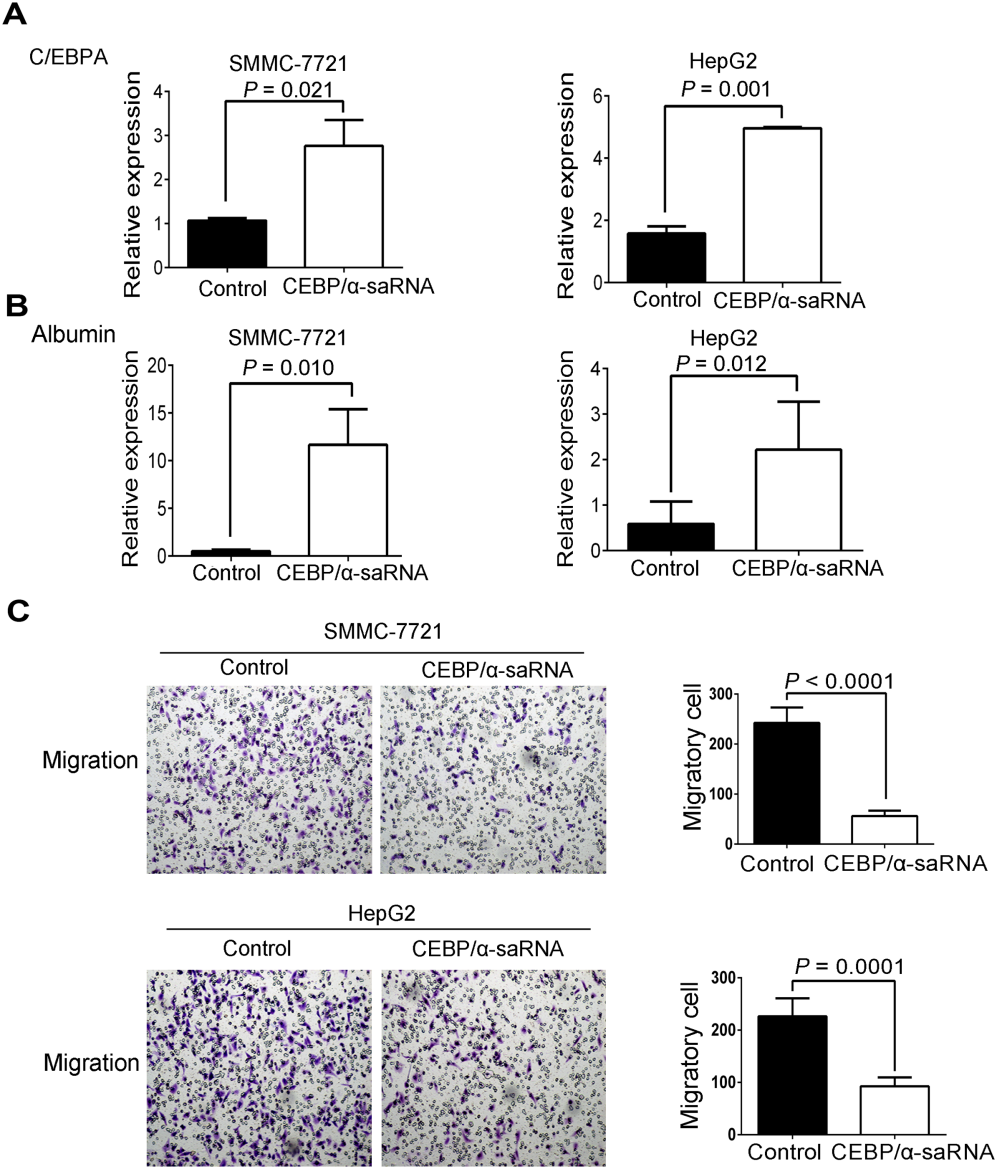
Transfection of C/EBPα-saRNA in SMMC-7721 and HepG2 cells resulted in inhibition of migration. (A–B) Gene expression of C/EBPα (A) and albumin (B) in SMMC-7721 and HepG2 cells transfected with C/EBPα-saRNA or scramble-saRNA as the control for 48 h. (C) Transwell assays show reduced migration of SMMC-7721 and HepG2 cells transfected with C/EBPα-saRNA compared with the control after 18 h. Magnification, 100×. Data represent mean±SD.

**Fig 3:**
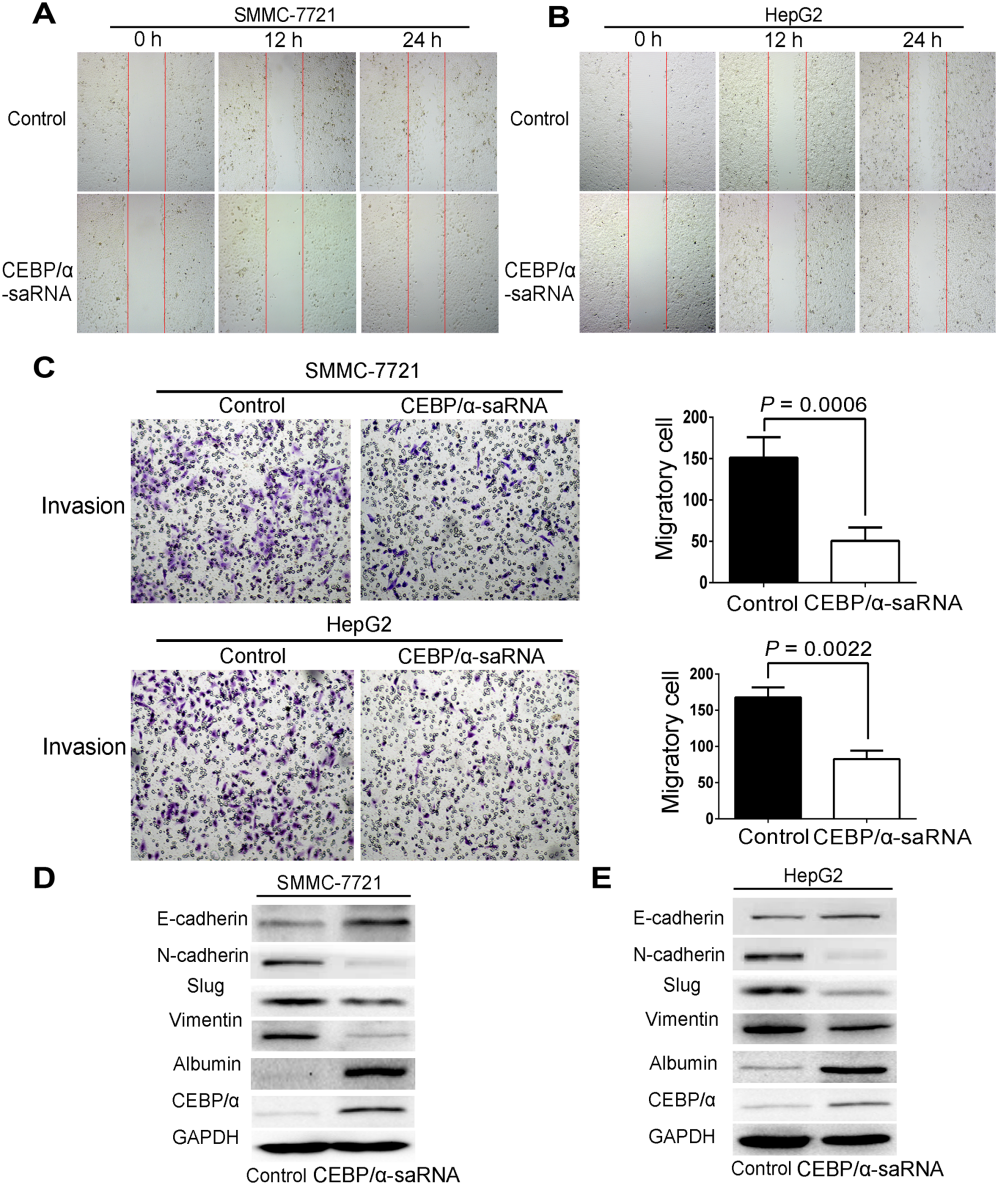
Transfection of C/EBPα-saRNA in hepatoma cells inhibited migration, invasion and EMT. (A-B) Wound healing assays of SMMC-7721 (A) and HepG2 (B) cells transfected with scramble-saRNA or C/EBPα-saRNA at 0 h, 12 h, and 24 h. Magnification, 40×. (C) Transwell assays show suppressed invasion of cells transfected with C/EBPα-saRNA compared with scramble-saRNA as the control after 24 h. Magnification, 100×. (D-E) Western blot analysis shows decreased N-cadherin, Slug, and Vimentin and up-regulation of E-cadherin, albumin, and C/EBPα in hepatoma cells transfected with C/EBPα-saRNA compared with the control. Data represent mean±SD.

To further explore the effect of C/EBPα-saRNA in hepatoma cells, we next performed *in vitro* analyses in C/EBPα-saRNA-transfected hepatoma cells. Both C/EBPα and albumin transcripts increased by at least two-fold in both SMMC-7721 and HepG2 cells, and albumin raised over ten-fold in SMMC-7721 (Fig 2A and 2B). Transwell migration assays (Fig 2C) firstly showed that C/EBPα-saRNA transfection resulted in significantly decreased migration of both SMMC-7721 and HepG2 cells. Wound healing assays were subsequently determined and revealed the consistent results (Fig 3A and 3B). Furthermore, transwell invasion assays indicated that invasive ability of the hepatoma cells was also inhibited when transfected with C/EBPα-saRNA (Fig 3C).

EMT is a biologic process that enables a polarized epithelial cell to undergo multiple biochemical changes [15]. The changes enable the cells to assume the functions of a mesenchymal cell phenotype. These functions are related to the mechanism of tumor metastasis [16]. Thus, we examined whether transfection of C/EBPα-saRNA in HepG2 and SMMC-7721 cells would affect EMT. C/EBPα and albumin were firstly determined as an increase level by western blot. It revealed that a significant reduction of mesenchymal markers, such as N-Cadherin, Slug, and Vimentin, and upregulation of E-Cadherin, an epithelial cell junction protein, in cells transfected with C/EBPα-saRNA compared to the scramble-saRNA (Fig 3D and 3E). These findings suggest that transfection of C/EBPα-saRNA may prevent liver tumor formation and hepatoma cell metastasis by inhibition of EMT.

### Transfection of C/EBPα-saRNA suppresses EGFR and β-catenin

**Fig 4:**
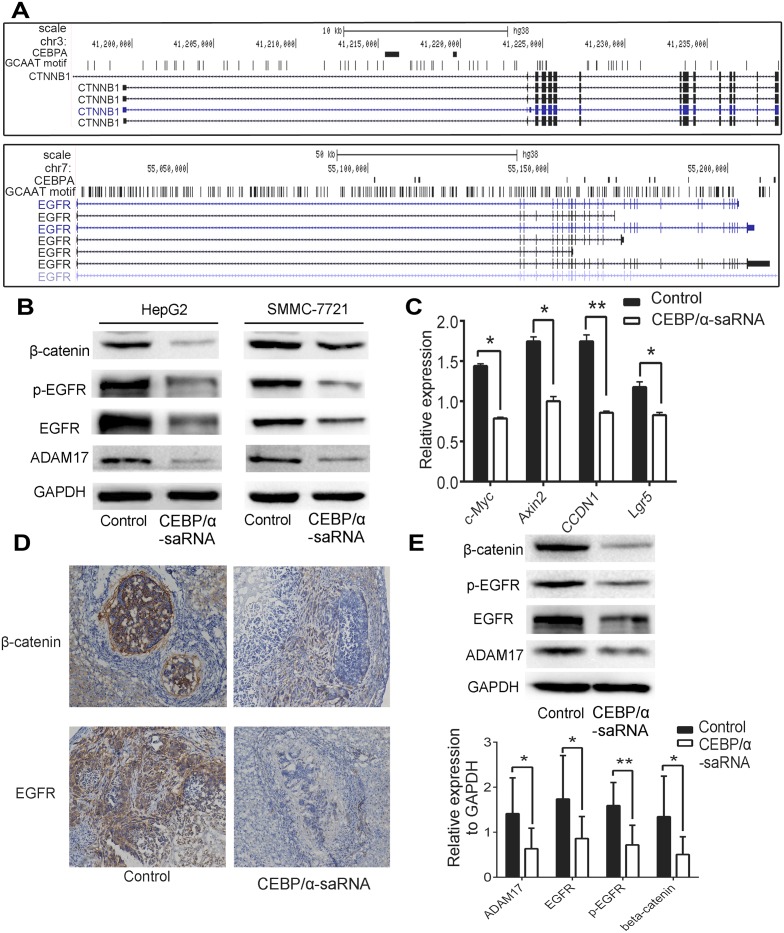
Transfection of C/EBPα-saRNA inhibits metastasis by targeting EGFR/β-catenin signaling pathway. (A) C/EBPα binding sites in EGFR and β-catenin genomic regions. (B) Western blot shows decreased ADAM17, EGFR protein and phosphorylation levels and decreased β-catenin in hepatoma cells transfected with C/EBPα-saRNA. (C) Reduced gene expression of c-Myc, Axin2, CCDN1, and Lgr5 in C/EBPα-saRNA transfected HepG2 cells compared with scramble-saRNA transfected as the control. (D) Immunohistochemistry analysis of EGFR, phosphorylation of EGFR, and β-catenin in liver tissues. (E) Western blot shows inhibited ADAM17, EGFR protein and phosphorylation levels and suppressed β-catenin in liver tissue of C/EBPα-saRNA-dendrimer-treated mice (n = 9).

Previous analysis of the gene expression profile of a panel of 84 liver cancer-specific genes in C/EBPα-saRNA-transfected HepG2 cells showed that most tumor genes including CTNNB1 (encoding β-catenin) and EGFR involved in EMT were down-regulated [14]. Evidences indicated that EMT was closely related to EGFR and β-catenin. EGF/EGFR axis triggered EMT in cholangiocarcinoma cells [17], and β-catenin targeted EMT to promote metastasis in colorectal cancer cells [18]. A ChIP-Seq analysis of EGFR and β-catenin showed one or more C/EBPα binding sites within their promoter regions (Fig 4A). Moreover, in C/EBPα-saRNA-transfected cells, we observed expression of EGFR, phosphorylation of EGFR, ADAM17 and β-catenin and found a strong reduction in protein level (Fig 4B). We next detected genes downstream of β-catenin and found that c-Myc, Axin2, CCDN1, and Lgr5 were down-regulated in C/EBPα-saRNA-transfected cells compared with scramble-saRNA-transfected cells (Fig 4C), indicating that C/EBPα-saRNA suppressed the EGFR/β-catenin pathway. To verify the results in hepatoma cells, liver tissues of nude mouse were then determined. Immunohistochemistry analysis revealed that expression of EGFR and β-catenin decreased in C/EBPα-saRNA-dendrimer-treated mouse (Fig 4D). ADAM17, EGFR, phosphorylation of EGFR and β-catenin were also down-regulated in treated mouse liver tissue by western blot (Fig 4E).

## Discussion

HCC is the most common primary malignancy of the liver [19]. Tumor metastasis is the cause of poor prognosis after hepatectomy, and thus high mortality of HCC [20].Several molecular pathways and regulatory genes have been implicated in HCC metastasis; however, the precise mechanisms remain elusive. Complicated mechanism or targets partly associated with etiology may result in the metastasis. C/EBPα is a nuclear transcription factor that functions in the regulation of tumor metastasis [21]. A saRNA oligonucleotide was used to increase the expression of C/EBPα in our study. It revealed that C/EBPα-saRNA-dendrimer treatment significantly inhibited both intrahepatic and distant metastasis and prolonged survival time in liver orthotopic xenograft tumor model. Moreover, liver injury was protected and liver function was improved in the treated mice. To verify these inhibitory effects *in vitro*, C/EBPα-saRNA was transfected to hepatoma cells. We found that expression of C/EBPα and albumin increased, and cell migration and invasion were inhibited. Inhibition of EMT and suppression of EGFR/β-catenin signaling were observed in C/EBPa-saRNA-transfected cells. It suggested that C/EBPα-saRNA successfully inhibits HCC metastasis by inhibiting EGFR/β-catenin signaling pathway mediated EMT *in vitro* and *in vivo*.

C/EBPα plays an essential role in the regulation of tumor metastasis. Combination of C/EBPα and promoter in miR-100 could suppress tumor metastasis by targeting ZBTB7A in gastric cancer [10]. As a tumor suppressor, activation of C/EBPα regulated miR-122 expression in HCC metastasis and invasion [22]. It is a mature method to design a saRNA oligonucleotide to increase the expression of a target gene [23,24]. C/EBPα-saRNA was previously successfully generated and shown to play an essential role in suppression of hepatocarcinogenesis [14]. Our results suggested that transfection of C/EBPα-saRNA, increased the expression of C/EBPα, inhibited liver tumor metastasis. It is consistent with previous reports that showed tumor suppressor activity of C/EBPα in head and neck squamous cell carcinoma [9], HCC [22], and pancreatic cancer [25].

Accumulating evidence has shown that metastasis is associated with EMT in a number of tumors [26–28]. Further, studies have demonstrated that EMT is a crucial event in HCC and associated metastasis, resulting in enhanced malignancy of HCC [29,30]. Higashikawa et al. [31] observed that Snail-induced EMT by down-regulation of p63 via suppression of C/EBPα-dependent transcription, leading to acquisition of an invasive phenotype and down-regulation of E-cadherin in human squamous cell carcinomas. These data identified the C/EBPα, a member of the C/EBP family of transcription factors, was observed in EMT phenotype cells, which exhibited invasive activity *in vitro*. Our results indicated that transfection of C/EBPα-saRNA could suppress migration and invasion of hepatoma cells, and high expression of E-cadherin and reduction of N-cadherin, Slug, and Vimentin in C/EBPα-saRNA transfected cells. Thus, C/EBPα acts as an important role in inhibition of metastasis by mediating EMT.

Tumor cells undergoing EMT express specific factors, including cytokines and chemokines, such as TGF-β, FGF, and EGF [32]. EGFR is aberrantly expressed or deregulated in tumors through the induction of EMT, playing crucial roles in metastatic progression [33], especially in cholangiocarcinoma [17] and HCC [34]. In a liver cancer pathway gene expression profile analysis of HepG2 cells transfected with C/EBPα-saRNA, EGF, EGFR, ADAM17 and β-catenin involved in EMT were down-regulated [14]. In the current study, we also observed down-regulation of ADAM17, EGFR, phosphorylation of EGFR, and β-catenin in C/EBPα-saRNA-transfected cells. It verified that ADAM17 caused the shedding of EGF, and indicating that ADAM17 might regulate signaling of EGFR. The EGF/EGFR axis triggers EMT in cholangiocarcinoma cells by inducing scatter of cholangiocarcinoma cells, as well as nuclear translocation of β-catenin [17]. Wnt pathway activated by growth factors such as EGF also induce β-catenin dissociation from the adherent junction complex, translocation into the nucleus, and activation of target genes such as c-Myc and involved in the EMT[35,36]. In order to investigate the association of C/EBPα and EGFR/β-catenin, we identified C/EBPα binding sites within the promoter regions of EGFR and β-catenin genes. It revealed that down-regulation of genes associated with β-catenin, including c-Myc, Axin2, CCDN1, and Lgr5 in C/EBPα-saRNA-transfected cells. Several studies showed that c-Myc [37], Axin2 [38], and Lgr5 [39], as downstream targets of β-catenin, induced an EMT phenotype in tumors. Here, we demonstrate an inhibition of EMT due to down-regulation of EGFR and β-catenin following C/EBPα-saRNA transfection.

In summary, our data demonstrated an inhibitory effect of C/EBPα-saRNA on metastasis *in vitro* and *in vivo*. We also confirmed the effect of C/EBPα-saRNA in promoting liver function in a liver orthotopic xenograft tumor model, and inhibiting migration and invasion in hepatoma cells. It demonstrated that C/EBPα-saRNA downregulated the EGFR/β-catenin signaling pathway, which inhibited EMT. Our findings help further the link between C/EBPα and tumor metastasis, and our data presented here may offer a new approach in HCC metastasis therapy.
